# A Genome-Wide Association Study of a Biomarker of Nicotine Metabolism

**DOI:** 10.1371/journal.pgen.1005498

**Published:** 2015-09-25

**Authors:** Anu Loukola, Jadwiga Buchwald, Richa Gupta, Teemu Palviainen, Jenni Hällfors, Emmi Tikkanen, Tellervo Korhonen, Miina Ollikainen, Antti-Pekka Sarin, Samuli Ripatti, Terho Lehtimäki, Olli Raitakari, Veikko Salomaa, Richard J. Rose, Rachel F. Tyndale, Jaakko Kaprio

**Affiliations:** 1 Department of Public Health, University of Helsinki, Helsinki, Finland; 2 Institute for Molecular Medicine FIMM, University of Helsinki, Helsinki, Finland; 3 National Institute for Health and Welfare, Helsinki, Finland; 4 Institute of Public Health and Clinical Nutrition, University of Eastern Finland, Kuopio, Finland; 5 Wellcome Trust Sanger Institute, Cambridge, United Kingdom; 6 Department of Clinical Chemistry, Fimlab Laboratories, Tampere, Finland; 7 Department of Clinical Chemistry, University of Tampere School of Medicine, Tampere, Finland; 8 Department of Clinical Physiology and Nuclear Medicine, Turku University Hospital, Turku, Finland; 9 Research Centre of Applied and Preventive Cardiovascular Medicine, University of Turku, Turku, Finland; 10 Department of Psychological and Brain Sciences, Indiana University, Bloomington, Indiana, United States of America; 11 Campbell Family Mental Health Research Institute, CAMH, and Departments of Pharmacology & Toxicology and Psychiatry, University of Toronto, Toronto, Canada; Yale School of Medicine, UNITED STATES

## Abstract

Individuals with fast nicotine metabolism typically smoke more and thus have a greater risk for smoking-induced diseases. Further, the efficacy of smoking cessation pharmacotherapy is dependent on the rate of nicotine metabolism. Our objective was to use nicotine metabolite ratio (NMR), an established biomarker of nicotine metabolism rate, in a genome-wide association study (GWAS) to identify novel genetic variants influencing nicotine metabolism. A heritability estimate of 0.81 (95% CI 0.70–0.88) was obtained for NMR using monozygotic and dizygotic twins of the FinnTwin cohort. We performed a GWAS in cotinine-verified current smokers of three Finnish cohorts (FinnTwin, Young Finns Study, FINRISK2007), followed by a meta-analysis of 1518 subjects, and annotated the genome-wide significant SNPs with methylation quantitative loci (meQTL) analyses. We detected association on 19q13 with 719 SNPs exceeding genome-wide significance within a 4.2 Mb region. The strongest evidence for association emerged for *CYP2A6* (min p = 5.77E-86, in intron 4), the main metabolic enzyme for nicotine. Other interesting genes with genome-wide significant signals included *CYP2B6*, *CYP2A7*, *EGLN2*, and *NUMBL*. Conditional analyses revealed three independent signals on 19q13, all located within or in the immediate vicinity of *CYP2A6*. A genetic risk score constructed using the independent signals showed association with smoking quantity (p = 0.0019) in two independent Finnish samples. Our meQTL results showed that methylation values of 16 CpG sites within the region are affected by genotypes of the genome-wide significant SNPs, and according to causal inference test, for some of the SNPs the effect on NMR is mediated through methylation. To our knowledge, this is the first GWAS on NMR. Our results enclose three independent novel signals on 19q13.2. The detected *CYP2A6* variants explain a strikingly large fraction of variance (up to 31%) in NMR in these study samples. Further, we provide evidence for plausible epigenetic mechanisms influencing NMR.

## Author Summary

Nicotine metabolism rate significantly varies between individuals and affects smoking behavior. Individuals with fast nicotine metabolism typically smoke more and thus have a greater risk for smoking-induced diseases. Further, the efficacy of smoking cessation pharmacotherapy is dependent on nicotine metabolism rate. Twin and family studies have shown that genes influence nicotine metabolism; however, only a minor fraction of variance in inter-individual differences in nicotine metabolism is accounted for by known reduced activity variants in *CYP2A6*, the main metabolic enzyme for nicotine. Here we utilized a biomarker of nicotine metabolism (nicotine metabolite ratio, NMR) in a genome-wide association study of three Finnish cohorts to identify novel genetic variants influencing nicotine metabolism rate. Our results enclose three independent novel signals in *CYP2A6*. The detected variants explain a strikingly large fraction of variance (up to 31%) in NMR in the study samples. A genetic risk score constructed using the independent signals predicts smoking quantity in two independent Finnish samples. Further, we enclose evidence for plausible epigenetic mechanisms influencing NMR. With the advent of other nicotine delivery devices than tobacco, such as e-cigarettes, the need to understand the long-term consequences and action mechanisms of nicotine and its metabolism are of high public health relevance.

## Introduction

Nicotine is a neuro-stimulant with high addiction potential [1]. Similar to other drugs causing dependence, nicotine increases dopamine levels in the nucleus accumbens and activates the mesolimbic brain reward pathway [2]. Nicotine metabolism rate varies significantly between individuals and is strongly correlated to total nicotine clearance thus altering nicotine levels from a given intake [3]. Smokers are known to titrate their nicotine levels via cigarette consumption, number and volume of puffs, and depth of inhalation, to achieve and maintain desired levels; thus, nicotine clearance rate influences smoking behavior [4]. Individuals with fast metabolism typically smoke more, are less likely to succeed in quitting, and are more prone to nicotine dependence (ND) [5]. Consequently, those who smoke more have a greater risk for smoking-induced diseases [6]. Beyond the evident toxic effects of tobacco smoke, there is increasing evidence that, in addition to the known acute effects of nicotine [7], chronic nicotine exposure as such may also increase the risk of cancer by multiple mechanisms [8]. With the advent of other nicotine delivery devices than tobacco, such as e-cigarettes, the need to understand the long-term consequences and action mechanisms of nicotine and its metabolism are of high public health relevance.

A member of the cytochrome P450 family, CYP2A6, is the main metabolic enzyme for nicotine accounting for up to 80% of nicotine clearance [9]. A large number of distinct *CYP2A6* (ENSG00000255974) alleles have been identified, including SNPs, duplications, deletions, and conversions (www.cypalleles.ki.se/cyp2a6.htm). *CYP2A6* variations have been phenotypically grouped as slow (<50% of activity), intermediate (80% of activity), and normal (100% of activity) metabolizers. Another member of the cytochrome P450 family, CYP2B6, has an approximately 10% catalytic efficiency of the CYP2A6 enzyme *in vitro* in nicotine c-oxidation, and may play a minor role in nicotine clearance at higher nicotine levels [10] or in the absence of functional CYP2A6. While *CYP2A6* is expressed primarily in the liver, *CYP2B6* (ENSG00000197408) is expressed at higher levels in the brain, where it may influence localized metabolism of nicotine [11, 12]. Cytochrome P450 drug metabolizing enzymes are rarely highlighted in genome-wide association studies (GWAS) as the allele frequencies of the functional variants are low in most populations [13]. However, in 2010 a very large GWAS meta-analysis of smoking quantity revealed associations of *CYP2A6* and *CYP2B6* with SNPs that are in strong linkage disequilibrium (LD) with known functional variants [14].

Nicotine metabolism involves multiple steps and several enzymatic pathways. Up to 75% of nicotine is converted to cotinine mainly by CYP2A6, 15% of nicotine is metabolized through other metabolic pathways, and a minor fraction (10–15%) is excreted to urine unchanged. The majority of cotinine is further converted to 3-hydroxycotinine exclusively by CYP2A6; up to 40% is excreted to urine as 3-hydroxycotinine while 10% is further metabolized into 3-hydroxycotinine-glucuronide by UGT enzymes prior to excretion. Approximately 15% of cotinine is converted to cotinine-glucuronide by UGT enzymes, and the remaining is metabolized through other pathways. [9]

Cotinine is a relatively stable compound with a half-life of 15–20h, and is superior as a biomarker of nicotine intake compared to self-reported smoking quantity (cigarettes per day, CPD) [15]. The ratio of 3-hydroxycotinine/cotinine (*i*.*e*. nicotine metabolite ratio, NMR) is an established biomarker of CYP2A6 activity, as well as nicotine metabolism rate, and it correlates strongly with total nicotine clearance [3]. Twin studies suggest an important genetic contribution to nicotine metabolism, measured using the nicotine metabolite ratio. Swan and colleagues reported that the estimated additive genetic effects on plasma NMR were 0.67 (95% CI 0.56–0.76), dropped to 0.61 (95% CI 0.48–0.71) after adjusting for non-genetic covariates, and further reduced to 0.49 (95% CI 0.33–0.63) when *CYP2A6* was adjusted for [16], suggesting that known *CYP2A6* variants identified at the time accounted for approximately 15–20% of NMR heritability.

In addition to the strong effect of *CYP2A6* and other genetic influences, NMR is also influenced by various demographic and hormonal factors. According to a recent study [17], factors affecting NMR include ethnicity, likely due to the varying frequency of reduced activity *CYP2A6* variants among different ethnicities [13]. Other affecting factors include sex, hormone replacement therapy, and use of estrogen containing contraceptive pills, all related to *CYP2A6* being induced by estrogen [18], as well as body mass index (BMI), alcohol consumption, and cigarette consumption [17]. Altogether these above mentioned factors were estimated to account for approximately 8% of inter-individual variance in NMR [17].

In addition to significantly affecting smoking behavior, nicotine clearance rate has been shown to contribute to the efficacy of cessation pharmacotherapy in various retrospective studies [19–21]. In a recent randomized prospectively NMR-stratified placebo-controlled clinical trial, varenicline (a prescription medication for smoking cessation) was more efficacious for normal metabolizers compared to nicotine patch [22]. Nicotine patch was equally effective in slow metabolizers with less side-effects than in varenicline treatment, suggesting that tailored pharmacotherapy, i.e. stratifying on NMR level, can be one approach for improving smoking cessation rates [22]. This first clinical trial to take nicotine metabolism rate into account is a landmark paper highlighting the importance of the metabolic component cigarette smoking and use of other nicotine products.

GWAS using metabolites measured from serum have been highly successful in identifying underlying genes [23, 24], highlighting the power of informative phenotypes. Our objective was to utilize NMR, a genetically informed biomarker of nicotine metabolism, in a GWAS meta-analysis of cotinine-verified (≥10ng/ml) current smokers from three Finnish cohorts to identify novel genetic variants influencing nicotine metabolism rate. In Caucasians, with up to 90% of individuals being normal metabolizers, a minor fraction of variance in inter-individual differences in nicotine metabolism is accounted for by known reduced activity *CYP2A6* variants. Thus, we expected that other contributing factors, such as other genes, but also novel regulators of CYP2A6 action or expression, including epigenetic mechanisms, are bound to exist. Our data highlighted novel genetic and epigenetic influences on NMR, deepening our understanding on factors influencing nicotine metabolism and providing valuable guidelines for further focused studies.

## Material and Methods

### Study samples

#### FinnTwin12

FinnTwin12 is a population-based longitudinal study of five consecutive birth cohorts (1983–1987) of Finnish twins, designed to examine genetic and environmental determinants of health-related behaviors [25, 26]. The study has a two-stage sampling design. The first-stage is an epidemiological investigation, with four waves of data collection (at ages 12, 14, 17, and at early adulthood) providing data on approximately 2700 families with twins. The second-stage is an intensive assessment of a sub-sample nested within the epidemiological study. Most of the sub-sample is selected at random, but this random sample is then enriched with twins from families with a history of elevated familial risk for alcoholism. The 1295 subjects of the intensive sample have DNA available while serum was available on 780 subjects. Among this sample, cotinine and 3-hydroxycotinine were measured from all self-identified current smokers. A total of 211 subjects (55 monozygotic (MZ) individuals (one co-twin from each MZ pair), 80 dizygotic (DZ) individuals from 40 full DZ pairs and additional 76 DZ individuals (one co-twin from each DZ pair)) with cotinine above 10ng/ml and 1000Genomes imputed genome-wide genotype data available were included in the NMR GWAS analyses. Genome-wide Infinium 450k Methylation BeadChip data (from whole blood DNA) were available on 157 of the NMR GWAS subjects.

#### FinnTwin16

FinnTwin16 is a population–based longitudinal study of five consecutive birth cohorts (1975–1979) of Finnish twins and their families [25, 26]. Initially, 3065 families (6130 twins) were contacted, with a 91% response rate. Questionnaire assessments including detailed alcohol and smoking data were conducted on twins as they reached the ages of 16, 17, 18½, and young adulthood (ages 22–25). For an intensive study on predictors of alcoholism, both twin pairs with very similar alcohol use and twin pairs with dissimilar alcohol use were identified [27, 28]. Additionally, some randomly picked twin pairs functioning as a control group were selected. The 602 subjects of the intensive sample have DNA and serum available. Among this sample, cotinine and 3-hydroxycotinine were measured from all self-identified current smokers, and 174 subjects (40 MZ individuals (one co-twin from each MZ pair), 76 DZ individuals from 38 full DZ pairs, and additional 58 DZ individuals (one co-twin from each DZ pair)) with cotinine above 10ng/ml and 1000Genomes imputed genome-wide genotype data available were included in the NMR GWAS analyses. Genome-wide Infinium 450k Methylation BeadChip data (from whole blood DNA) were available on 14 of the NMR GWAS subjects.

#### The Young Finns Study (YFS)

The Young Finns Study (YFS) is a large follow-up study of cardiovascular risk factors from childhood to adulthood [29]. In total, 3596 children and adolescents aged 3–18 from all around Finland participated in the baseline study in 1980; they were followed up after, 3, 6, 9, 12, 21, 27, and 30 years, with comprehensive risk factor assessments, including smoking status and alcohol use. Cotinine and 3-hydroxycotinine were measured from all self-identified current smokers with available serum samples, and 714 subjects with cotinine above 10ng/ml and 1000Genomes imputed genome-wide genotype data available were included in the NMR GWAS analyses. For 166 subjects with repeated serum samples as current smoker, i.e. longitudinal metabolite data the NMR was assessed at the time of heaviest regular smoking (i.e. when cotinine was highest), although NMR can be used in light regular smokers [30].

#### The FINRISK

The FINRISK studies are population surveys of the Finnish adult population conducted every five years since 1972 [31], examining risk factors of chronic diseases. The FINRISK2007 study consists of 6258 men and women aged 25–74 years drawn from the national population register in five large geographical areas in Finland at the end of 2006. The sample was stratified by sex, 10-year age category, and area. FINRISK2007 includes a smoking-specific questionnaire given to a subsample of ever-smokers who attended an in-person clinical examination and blood draw. Cotinine and 3-hydroxycotinine were measured among self-identified current smokers and recent quitters as part of an analysis of the validity of self-reported smoking status. A total of 419 subjects with cotinine above 10ng/ml and 1000Genomes imputed genome-wide genotype data available were included in the NMR GWAS analyses. For scrutiny of linkage disequilibrium (LD) patterns and allele frequencies, as well as for Genetic Risk Score analyses, additional non-overlapping samples from FINRISK cohorts 1992, 1997, 2002, and 2007 (N = 19857 after individuals with pi-hat>0.4 were excluded; cotinine and 3-hydroxycotinine not available in these) with 1000Genomes imputed genome-wide genotype data available were used.

#### The NAG-FIN

The NAG-FIN sample was ascertained from the Older Finnish Twin Cohort consisting of adult twins born in 1938–1957. Based on earlier questionnaires, twin pairs concordant for ever-smoking were recruited along with their family members (mainly siblings) for the Nicotine Addiction Genetics (NAG) consortium [32]. Data collection took place in 2001–2005. A total of 747 families including 2193 subjects were assessed by DNA sample collection, structured psychiatric interview, and additional questionnaires, yielding detailed phenotypes of lifetime smoking behavior (including initiation, quantity, and cessation). For the Genetic Risk Score analyses 2054 subjects from 745 families (including 141 MZ twins (one co-twin from each MZ pair), 856 DZ individuals from 428 full DZ pairs, additional 147 DZ individuals (one co-twin from each DZ pair), 50 individuals of unknown zygosity, and 860 other family members (mainly siblings)) with 1000Genomes imputed genome-wide genotype data were used. Serum samples for cotinine and NMR analyses were not collected.

### Ethics permissions

Written informed consent, according to the current edition of the Declaration of Helsinki, was obtained from all subjects who were interviewed and/or gave DNA samples before the beginning of the studies. The collection of blood samples followed the recommendations given in the Declaration of Helsinki and its amendments. For the FinnTwin12, FinnTwin16, and NAG-FIN studies, data collection has been approved by the hospital district of Helsinki and Uusimaa, the ethics committee for epidemiology and public health (HUS-113-E3-01, HUS-346-E0-05, HUS 136/E3/01). FinnTwin12 and FinnTwin16 have also been approved by the IRB of Indiana University at Bloomington, Indiana, while NAG-FIN was also approved by the IRB of Washington University (St. Louis). Young Finns Study data collection has been approved by the hospital district of Southwest Finland ethics committee (ETMK: no. 88/180/2010). The ethics approvals for FINRISK have been obtained from the Coordinating Ethics Committee of Helsinki and Uusimaa Hospital District.

### Phenotypes and covariates

Cotinine and 3-hydroxycotinine phenotypes were measured from frozen serum samples using liquid chromatography/tandem mass spectrometry (University of Toronto, prof. Rachel Tyndale’s laboratory) for the FinnTwin12, FinnTwin16, and YFS samples, as previously described [33], and using gas chromatograph-mass spectrometer (at the National Institute for Health and Welfare, Helsinki, Finland) for the FINRISK2007 sample, as previously described [34]; these assays for NMR were demonstrated to be fully concordant (R^2^ = 0.93, P<0.0001) [35]. Cotinine threshold of ≥10ng/ml was applied to restrict the analyses to regular smokers.

Several potential factors influencing on NMR (sex, age, BMI, smoking quantity, alcohol consumption, genotyping batch) [17] were considered as covariates. Sex and BMI were associated with NMR (p<0.05) in the initial linear univariate regression models, and were selected as covariates for the study specific GWAS. Further, age was selected as a covariate due to the sampling design of the population-based samples. Neither smoking quantity, alcohol consumption, nor genotyping batch, were significant confounders (p>0.05) and thus were not included as covariates. Details of phenotype and covariate distributions in the three samples are presented in Table 1.

**Table 1:** Phenotype and covariate proportions (%) and means (min-max, SD) in the FinnTwin, Young Finns Study (YFS), and FINRISK2007 samples.

	FinnTwin (N = 385)	YFS (N = 714)	FINRISK2007 (N = 419)
% male	50%	54%	54%
Age	24 (21–30, 2.0)	30 (15–45, 8.6)	49 (25–74, 12.8)
BMI	24 (16–43, 4.0)	24 (16–46, 3.9)	27 (16–50, 5.0)
CPD	11 (1–40, 5.9)	13 (1–64, 7.6)	13 (0–40, 8.4)
Cotinine (ng/ml)	182 (10–656, 122.5)	185 (11–577, 112.4)	170 (24–610, 94.7)
3-hydroxycotinine (ng/ml)	70 (1–275, 48.2)	77 (1–435, 55.0)	60 (4–293, 40.7)
NMR	0.4 (0.01–1.6, 0.22)	0.5 (0.01–1.7, 0.24)	0.4 (0.04–2.0, 0.21)

### Genotyping and imputation

Genotyping was performed with the Human670-QuadCustom Illumina BeadChip at the Welcome Trust Sanger Institute, and with the Illumina Human Core Exome BeadChip at the Welcome Trust Sanger Institute and at the Broad Institute of MIT and Harvard. Standard post-genotyping quality control thresholds were applied for SNPs (minor allele frequency (MAF) <0.01, SNP call rate <0.95, and Hardy Weinberg Equilibrium (HWE) p<1E-06). Further, subjects with a call rate <0.95 were excluded, and a sample heterozygosity test, as well as sex and Multidimensional Scaling (MDS) outlier checks were done. Pre-phasing of the data was done with SHAPEIT2 [36] and imputation with IMPUTE2 [37] using the 1000 Genomes Phase I integrated haplotypes (produced using SHAPEIT2) reference panel [38]. For data generated with the Human670-QuadCustom Illumina BeadChip the following post-imputation exclusion criteria were applied for SNPs: MAF<0.01, SNP call rate <0.95 (<0.99 for SNPs with MAF<0.05), HWE p<1E-06, and imputation info <0.4. For data generated with the Illumina Human Core Exome BeadChip the SNP exclusion criteria were otherwise identical, except that a threshold of minor allele count <2 was applied instead of a MAF cut-off. Further, the same sample quality thresholds as in post-genotyping quality control were applied. Quality controls and imputation for all Finnish GWAS data were done centrally at the Institute for Molecular Medicine, University of Helsinki, Helsinki, Finland.

### Statistical analyses

#### Twin modelling

Altogether 81 DZ twin pairs (32 same-sex and 49 opposite-sex) and 54 MZ twin pairs with cotinine (>10 ng/ml in both co-twins) and 3-hydroxycotine measures available in the FinnTwin12 and FinnTwin16 samples were used to estimate the heritability of NMR. Intraclass correlations were calculated and quantitative genetic modelling was used to estimate alternative variance components models in Mx [39]. We evaluated the presence of additive genetic (A), genetic effects due to dominance (D), shared environmental effects (C), and environmental effects not shared by twins (E).

#### Power analyses

We estimated the power of our GWAS meta-analysis sample to detect signals at the p<5E-08 significance threshold assuming an LD (r^2^) of 0.8 between the causal allele and the marker allele, as previously suggested [40]. To account for intraclass correlation within 78 DZ pairs included in the FinnTwin sample, we estimated the effective sample size (N_eff_) for these 156 DZ twins using the formula N_eff_ = N/(1 + (m ‐ 1)ICC), where N is the number of DZ twins (156 twins in our case), m is the number of observations in a group (2 in our case), and ICC is the intraclass correlation (0.26 estimated from FinnTwin data), resulting in an estimated effective sample size of 124 (a reduction of 32). Thus, the effective sample size in our meta-analysis was reduced from 1518 to 1486. We performed power analyses using the Genetic Power Calculator [41].

#### GWAS analyses

Prior to the analyses NMR was transformed using rank transformation to obtain a normal distribution. The transformation was employed with the rntransform() function available in the R statistical software package GenABEL [42], which replaces the median NMR value with the value zero and transforms the rest of the values so that they are N(0,1)-normally distributed around this value. NMR distributions before and after the transformation are shown in S1 Fig.

GWAS analyses were done using GEMMA (Genome-wide efficient mixed-model association) [43], separately for FinnTwin (FinnTwin12 and FinnTwin16 samples were pooled together), Young Finns Study, and FINRISK2007 cohorts. Allelic dosage data were used to account for genotype uncertainties. The genetic associations were acquired with a linear mixed model in which the rank transformed NMR was the dependent variable and the coded allele dose (represented by the posterior mean genotypes) was the independent variable. The model included age, sex, and BMI as covariates (fixed effects). In addition, population stratification and relatedness within the sample were accounted for by the covariance matrix of the random effect in the model. The covariance matrix was determined by a relatedness matrix, calculated from genome-wide genotype data and representing genetic similarity across individuals. An estimate of the genomic control inflation factor (λ) was calculated for all three cohorts with the estlambda() function of the R library GenABEL [42]. P-values below 5E-08 were considered as genome-wide significant.

#### GWAS meta-analysis

Meta-analysis was performed with the software META [44]. The fixed effects model, using the inverse variance method, was chosen as all cohorts had the same phenotype and measurement scale. Briefly, the cohort-specific beta estimates were summed together by weighting the cohort-specific beta estimates by the inverse of their variance in order to take into account the contribution of each cohort. In addition, the variances of the cohort-specific beta estimates were first multiplied by the cohort-specific genomic control λ estimates. A total of 8970401 SNPs were included in the meta-analysis (the number of SNPs in the cohort-specific GWAS analyses was 9087865 in FinnTwin, 8829569 in YFS, and 9050503 in FINRISK2007). The genomic control inflation factor (λ) in the meta-analysis was 1.027, while in cohort-specific GWAS they were 1.038 in FinnTwin, 1.016 in YFS, and 1.000 in FINRISK2007.

#### Conditional analyses

Genomic loci reaching genome-wide significance in the meta-analysis were further targeted with conditional analyses to estimate the number of independent signals. We ran association analyses separately in each of the three cohorts conditioning on our SNP with the lowest p-value, followed by a meta-analysis of the three cohorts. The next signal was identified from the conditional meta-analysis results, and included in the second round of conditional analyses. This process was repeated in an iterative fashion until no residual genome-wide significant signal (p<5E-08) remained.

After conditioning on the top-SNP (rs56113850), rs113288603 emerged as the second independent SNP. As this SNP showed no association in either the cohort-specific GWAS or in meta-analysis, we further scrutinized the plausible interplay between rs56113850 and rs113288603 within the largest of our cohorts (YFS), by testing how adding the other SNP or an interaction term (rs56113850*rs113288603) to the model affects the effect sizes.

#### Percentage of variance explained by the SNPs

Percentage of variance in rank transformed NMR explained by the independent SNPs, as well as the known *CYP2A6* alleles [CYP2A6*2 (= rs1801272), CYP2A6*9 (= rs28399433), CYP2A6*14 (= rs28399435), CYP2A6*18 (= rs1809810), CYP2A6*21 (= rs6413474), allele naming according to the CYP2A6 Allele Nomenclature (www.cypalleles.ki.se/cyp2a6.htm)) included in our 1000Genomes imputed data set, was estimated by running linear regression models separately in each of the three cohorts, with NMR as the dependent variable and age, sex, BMI, and SNPs as explanatory variables. The variance explained by a specific SNP or SNPs was acquired by subtracting the R^2^ from the model including only the covariates (age, sex, BMI) from the R^2^ of the model including also the SNP/SNPs of interest as explanatory variables.

#### Genetic risk score analyses

We constructed 3-SNP weighted genetic risk scores (wGRS) using the independent 19q13.2 SNPs from the meta-analysis (rs56113850, rs113288603, esv2663194) by summing the number of major alleles (0/1/2) for each of the three SNPs weighted by their estimated effect sizes obtained from the GWAS meta-analysis. In addition, a 4-SNP wGRS comprising of the four independent SNPs in FINRISK2007 (rs56113850, rs113288603, esv2663194, rs12461964) was constructed. We then tested whether the wGRSs predict smoking behavior using two independent Finnish samples. In the FINRISK sample (no overlap with the FINRISK2007 sample used in NMR GWAS), logistic regression was used to model current daily (N = 3138) vs. former (abstinence of ≥6 months; N = 2710) smoking, and hurdle regression was used to model smoking quantity among current smokers (quantitative CPD; N = 3138). In the family-based NAG-FIN sample, mixed effects logistic regression was used to model current daily (N = 816) vs. former (abstinence of ≥6 months; N = 833) smoking, and a mixed effects poisson regression was used to model smoking quantity among current smokers (quantitative CPD; N = 816). Odds ratios (ORs) obtained for current vs. former smoking depict whether it is more (OR>1) or less (OR<1) likely to fall into category of ‘former smoker’ as wGRS increases. Regression coefficients (betas) obtained for smoking quantity indicate the increase (positive beta) or decrease (negative beta) in CPD as wGRS increases. All analyses were adjusted for age, sex, and BMI, and families were assumed to form clusters in the NAG-FIN sample. Finally, a meta-analysis of the two samples was performed. Additionally, we utilized available questionnaire data of potential confounders of cessation. In a sensitivity analysis we excluded (i) 62 (7.4%) NAG-FIN former smokers who report quitting due to adverse health effects, and (ii) 188 (23%) current and 176 (21%) former smokers who reported having a DSM-IV (Diagnostic and Statistical Manual of Mental Disorders, 4^th^ edition) major depressive disorder diagnosis. For FINRISK we excluded 820 (26%) current smokers and 775 (29%) former smokers who either (i) reported having a diagnosis of cancer of cardiovascular disease (angina pectoris, heart failure, asthma, pulmonary emphysema, or bronchitis), (ii) reported being on a disability pension, or (iii) reported self-rated health as ‘poor’ or ‘very poor’. Further, we adjusted the analyses for alcohol use, a known confounder, i.e. factor associated with both NMR and smoking behavior (grams of ethanol consumed during the last week in FINRISK, and grams of ethanol consumed on average per week in NAG-FIN). We also ran additional wGRS analyses with an rs56113850*rs113288603 interaction term included as a covariate, to account for the detected interplay between these two SNPs. As four independent SNPs emerged in the FINRISK2007 GWAS sample, and the main wGRS sample was derived from FINRISK cohorts, we report results for the 4-SNP wGRS; however, the 3-SNP wGRS analyses yielded highly similar results.

#### Annotation

For annotation of the plausible role of the highlighted SNPs, we considered three alternative hypotheses: (A) SNPs directly affect the function of CYP2A6 enzyme, or are in LD with functional variants, resulting in changes in NMR, (B) DNA methylation mediates the effect of SNPs on NMR, and (C) NMR mediates the effect of SNPs on methylation. To test hypothesis A, the potential functional consequences of the associating SNPs were predicted using the Ensembl Variant Effect Predictor database (http://www.ensembl.org/index.html) (includes predictions PolyPhen and SIFT). LD patterns were estimated by using the ‘solid spine of LD’ method of the program Haploview [45] from GWAS data generated in the FINRISK 1992, 1997, 2002, and 2007 cohorts (N = 19857). Similarly, minor allele frequencies representative of the Finnish population were obtained from the FINRISK sample (N = 19857). To test hypotheses B and C we proceeded with methylation quantitative loci analyses and Causal Inference Test (CIT).

#### Methylation quantitative trait loci (meQTL) analyses

In order to test whether the 719 genome-wide significant SNPs affect methylation we performed meQTL analyses. Methylation probes targeting CpG sites within the 4.2 Mb target region (*i*.*e*. the region with genome-wide significant association signal) with 500 kb flanking regions were identified from the Infinium HumanMethylation450 BeadChip (Illumina) data available in 171 FinnTwin12 and FinnTwin16 subjects included in the NMR GWAS. Array preprocessing and normalization was performed using the Bioconductor package ‘minfi’ [46], with Stratified Quantile Normalization using the function PreprocessQuantile. Altogether 2268 methylation probes map to the 4.2 Mb target region; 844 of these were excluded as they have been reported as unreliable based on various criteria (mapping to multiple genomic locations and known repeat regions, containing SNPs, etc.) [47]. Variance in methylation levels was estimated in the remaining 1424 CpG sites; as we had a limited sample size (N = 171) and thus limited power to detect association, we restricted the meQTL analyses to 158 CpG sites with reasonable variance (interquartile range ≥0.05; S2 Fig). We constructed linear regression models of DNA methylation levels at each CpG site as the dependent variable and dosage of coded allele at each SNP as the explanatory variable while adjusting for age and sex, as previously suggested [48, 49]. To account for multiple testing we used the Benjamini and Hochberg method [50] and considered false discovery rate (FDR) corrected p-values below 0.05 as statistically significant. Five CpG sites mapped to *CYP2A6* in the genome-wide methylation data; however, we were unable to scrutinize these as four probes were filtered out based on the quality control criteria and the fifth probe had a low (0.02) interquartile range.

#### Causal Inference Test (CIT)

We proceeded with a CIT with the 16 CpG sites highlighted in the meQTL analyses to establish the direction of relationship between a causal factor (G, genotype), a potential mediator (M, methylation), and an outcome (Y, NMR). Briefly, the conditions for CIT are (1) G and Y are associated, (2) G is associated with M after adjusting for Y, (3) M is associated with Y after adjusting for G, and (4) G is independent of Y after adjusting for M [51]. We performed CIT using the R script provided by Millstein and colleagues [51]. This CIT R script gives a formal p-value for causal model (‘DNA methylation mediates the effect of SNPs on NMR’), reactive model (‘NMR mediates the effect of SNPs on methylation’), and a causal call based on the two p-values obtained. To account for multiple testing we considered FDR corrected p-values below 0.05 as statistically significant.

## Results

MZ and DZ twins of the FinnTwin12 and FinnTwin16 cohorts were used to provide heritability estimates for NMR. The intraclass correlation for NMR was 0.80 for MZ and 0.26 for DZ pairs. The pattern of correlations suggested that in addition to additive genetic effects, dominance effects may be present but shared environmental were unlikely to be present. The data were consistent with both AE and ADE models, but not the ACE model as the MZ correlation was much larger than twice the DZ correlation (in such a situation the C component will be zero). In the AE model, A effects accounted for 0.81 (95% CI 0.70–0.88) of variance in NMR. The ADE model fit difference was not statistically significant from the more parsimonious AE model (p = 0.087, Δχ^2^ = 2.94, Δdf = 1); in the ADE model A effects accounted for 0.20 (95%CI 0.00–0.85) and D effects for 0.62 (95%CI 0.00–0.88) of the variance in NMR.

According to the power analyses we had inadequate power to detect signals with rare SNPs (MAF <5%) unless they have very large effect sizes, but high power to detect signals with common SNPs (MAF >5%) that have medium to high effect sizes (beta>±0.6) (S1 Table).

**Fig 1:**
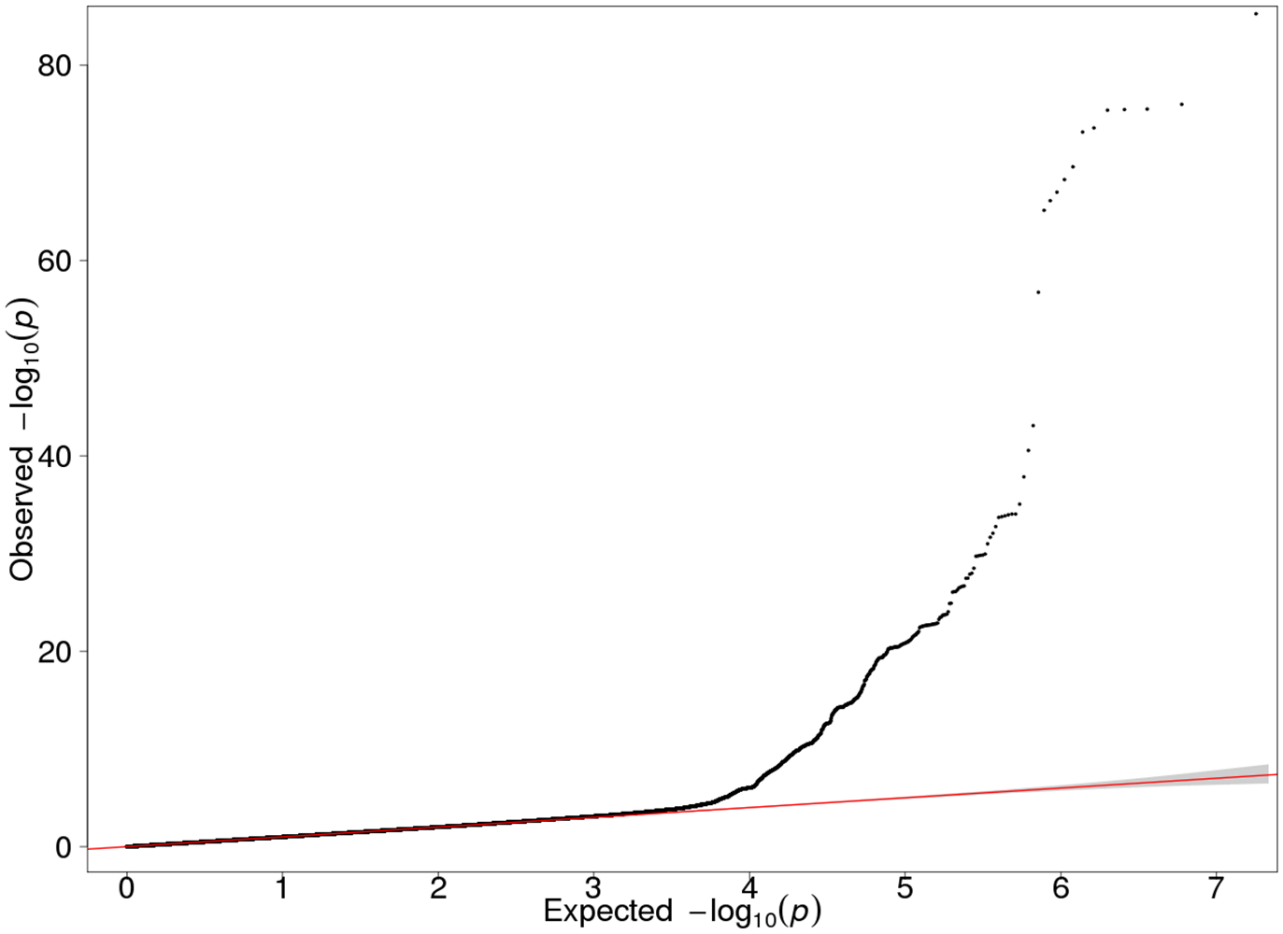
QQ plot of the GWAS meta-analysis results of the three Finnish cohorts (λ = 1.027).

**Fig 2:**
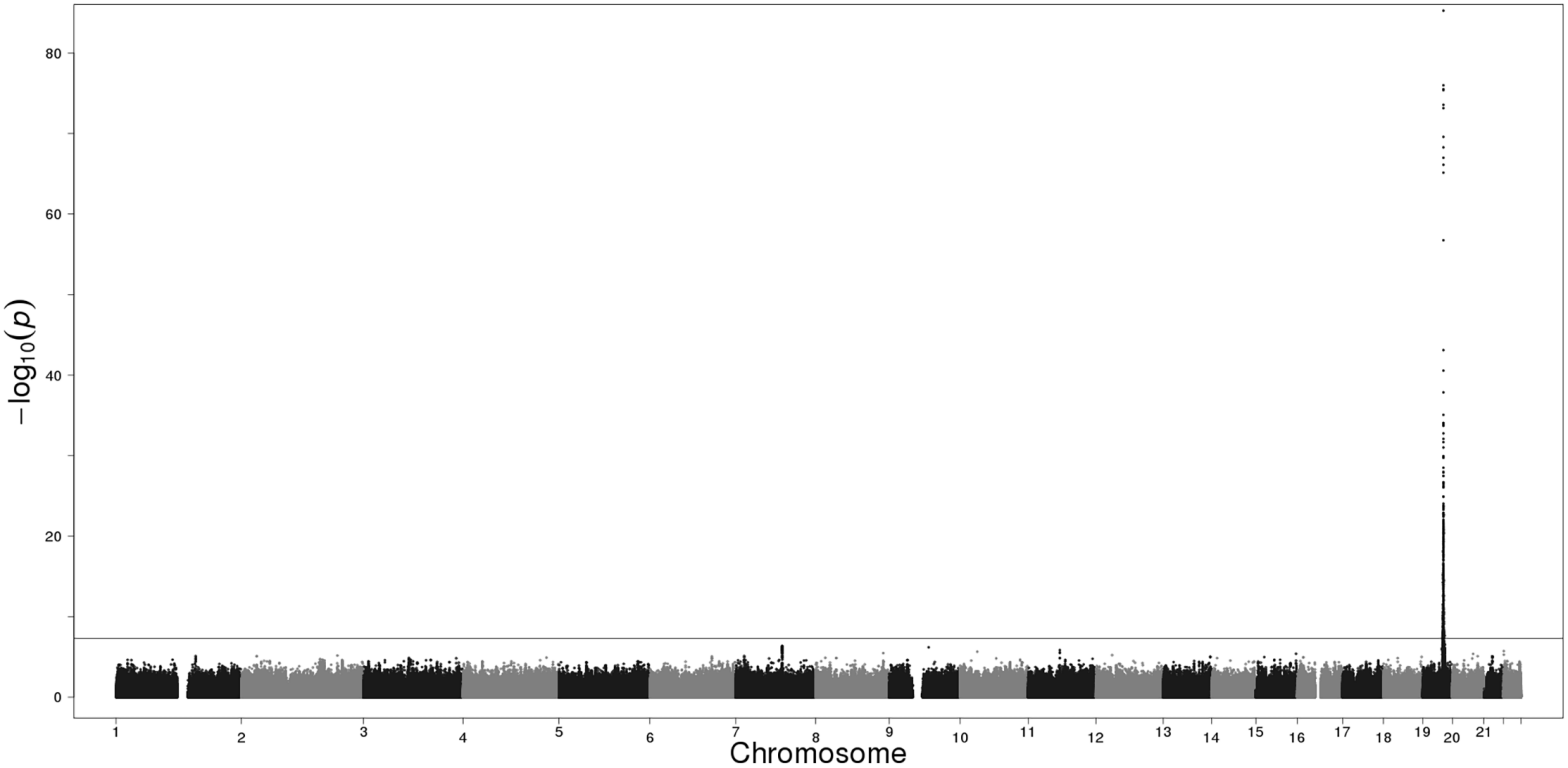
Manhattan plot of the GWAS meta-analysis results of the three Finnish cohorts. The horizontal line represents the genome-wide significance threshold (p<5E-08).

Each of the three Finnish cohorts independently showed genome-wide significant association on 19q13.2. In our GWAS meta-analysis 719 SNPs exceeded the genome-wide significance threshold within a 4.2 Mb region on 19q13.2 (chr19:39546965–43710562; according to GRCh37/hg19) (S2 Table). Manhattan and QQ plots are presented in Figs 1 and 2. The strongest evidence for association emerged for *CYP2A6* (minimum p = 5.77E-86 for rs56113850, in intron 4) (S3 Table). Other genes of relevance with genome-wide significant signals included *CYP2B6* (minimum p = 1.95E-24 for rs7260329, in intron 8), *CYP2A7* (ENSG00000198077) (minimum p = 1.43E-38 for rs28602288, *beta* = -0.48, -83C>T, within predicted promoter region), *EGLN2* (ENSG00000269858) (minimum p = 7.87E-16 for rs76443752, in intron 3), and *NUMBL* (ENSG00000105245) (minimum p = 1.40E-20 for rs4802082, in intron 5) (S2 Table).

**Fig 3:**
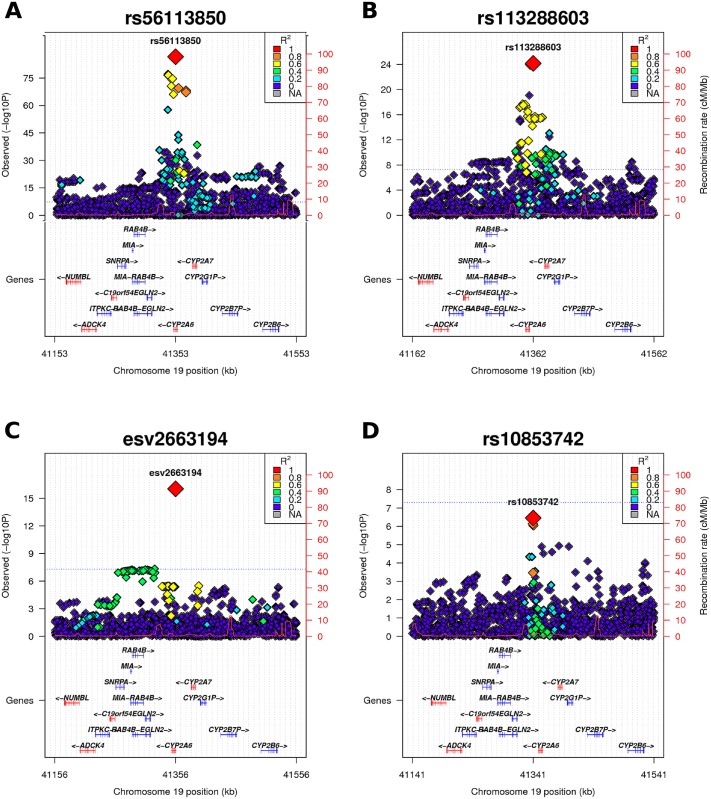
Regional plot of 19q13.2. (A) In the GWAS meta-analysis a minimum p-value of 5.77E-86 was obtained for rs56113850, in intron 4 of *CYP2A6*. (B) Analyses conditioned on the top-SNP (rs56113850) revealed rs113288603 as a second independent genome-wide significant signal (p_cond_ = 7.03E-25). (C) Analyses conditioned on the two independent SNPs (rs56113850, rs113288603) revealed esv2663194 as a third independent genome-wide significant signal (p_cond_ = 9.3E-17). (D) Analyses conditioned on the three independent SNPs yielded no additional signal exceeding the genome-wide significance threshold. All plots are generated with LocusTrack [52]. LD (R^2^) values were obtained from the FINRISK sample (N = 19857).

Conditional analyses of the 19q13.2 locus revealed three independent genome-wide significant signals tagged by rs56113850, rs113288603, and esv2663194 (Fig 3 and Table 2). The same independent SNPs emerged in all the three cohorts. A fourth independent signal (rs12461964) emerged in the conditional analysis of the FINRISK2007 sample; however, this was not seen in the meta-analysis of the three samples. All the independent SNPs are imputed, and are located either within *CYP2A6* or at most at a distance of 8 kb from the gene. For all the independent SNPs the minor allele decreases NMR, *i*.*e*. decreases nicotine clearance rate (Table 2). None of the independent SNPs have predicted functional effects.

**Table 2:** Detailed results for the most interesting signals on 19q13.2. First four rows represent the independent signals in the ranking order from the meta-analysis of conditional analyses. Last five rows represent known *CYP2A6* alleles present in our data. Distribution of NMR and rank transformed NMR among the different genotype groups of these nine SNPs in the YFS sample are presented in S6 Fig.

		FinnTwin			Young Finns Study			FINRISK2007			MAF		GWAS meta-analysis			
	Location with respect to *CYP2A6*	MAF	GWAS *beta* ^1^ (SD), p-value	% of variance explained	MAF	GWAS *beta* ^1^ (SD), p-value	% of variance explained	MAF	GWAS *beta* ^1^ (SD), p-value	% of variance explained	FINRISK^2^	EUR^3^	p-value	*beta* (coded allele)	*beta* (minor allele)	Estimated change in NMR^4^
rs56113850	C>T in intron 4	0.46	-0.61 (0.07), p = **2.78E-17**	14.4%	0.45	-0.70 (0.05), p = **4.56E-45**	22.8%	0.45	-0.60 (0.07), p = **8.76E-19**	17.0%	0.44	0.41	**5.77E-86**	-0.65	-0.65	-0.15
rs113288603 ^5^	C>T in intron 1	0.15	-0.01 (0.10), p = 0.89	<0.01%	0.15	0.07 (0.08), p = 0.34	0.14%	0.15	-0.03 (0.10), p = 0.78	0.04%	0.15	0.09	0.663	0.02	-0.02	-0.005
esv2663194	del of exons 1–2	0.03	0.78 (0.23), p = 8.71E-04	3.12%	0.04	1.18 (0.14), p = **4.14E-16**	7.95%	0.03	1.12 (0.24), p = 5.16E-06	4.72%	0.03	0.02	**3.34E-23**	1.08	-1.08	-0.25
rs12461964 ^6^	G>A in intron 1	0.48	-0.53 (0.07), p = **9.35E-15**	N/A ^7^	0.44	-0.63 (0.05), p = **2.25E-36**	N/A ^7^	0.45	-0.63 (0.06), p = **4.79E-22**	19.7%	0.45	0.51	**3.66E-76**	-0.61	-0.61	-0.14
CYP2A6*2 ^8^	L160H	<0.01	N/A ^9^	0.30%	0.02	1.45 (0.21), p = **7.62E-12**	6.12%	0.02	0.81 (0.33), p = 0.01	1.46%	0.01	0.03	**8.64E-13**	1.27	-1.27	-0.29
CYP2A6*9 ^10^	promoter (-48T>G)	0.14	0.45 (0.10), p = 1.06E-05	4.05%	0.12	0.64 (0.08), p = **1.74E-14**	6.98%	0.13	0.81 (0.11), p = **1.72E-13**	12.22%	0.11	0.07	**1.54E-30**	0.63	-0.63	-0.14
CYP2A6*14 ^11^	S29N	0.02	0.21 (0.24), p = 0.39	0.28%	0.02	0.33 (0.22), p = 0.14	0.19%	0.01	-0.11 (0.31), p = 0.73	0.02%	0.01	0.04	0.191	0.19	-0.19	-0.04
CYP2A6*18 ^12^	Y392F	0.02	-1.22 (0.30), p = 4.71E-05	2.93%	0.02	-0.84 (0.19), p = 1.39E-05	2.70%	<0.01	N/A ^9^	<0.01%	0.02	0.02	**5.24E-09**	-0.95	-0.95	-0.22
CYP2A6*21 ^13^	K476R	0.02	0.92 (0.23), p = 7.83E-05	2.63%	0.03	0.66 (0.15), p = 1.39E-05	2.65%	0.02	0.79 (0.28), p = 5.25E-03	1.90%	0.02	0.01	**1.39E-10**	0.75	-0.75	-0.17

MAF, minor allele frequency; GWAS, genome-wide association study; SD, standard deviation; EUR, 1000Genomes reference panel of individuals of European descent; N/A, not available;

^1^ *beta* reported for the coded allele;

^2^ MAF calculated among 19857 FINRISK individuals;

^3^ MAF reported for the EUR population at the Ensembl database;

^4^ change in NMR is estimated by multiplying SD of NMR (SD = 0.23 in the combined meta-analysis sample) by the effect size of the minor allele;

^5^ rs113288603 showed no association in the GWAS but was identified as an independent signal in analyses conditioned on the top-SNP (rs56113850);

^6^ identified as an independent SNP only in the FINRISK2007 GWAS sample;

^7^ rs12461964 was not an independent signal in FinnTwin or YFS, thus variance explained was not estimated;

^8^ rs1801272;

^9^ not included in the GWAS due to MAF<0.01;

^10^ rs28399433;

^11^ rs28399435;

^12^ rs1809810;

^13^ rs6413474.

A plausible interplay between the top-SNP rs56113850 and rs113288603 was detected. When analysed within the largest of our cohorts (YFS) the minor allelic effect size of rs113288603 increased from non-significant (*beta* = 0.05, p = 0.522) to highly significant (*beta* = -0.47, p = 1.32E-09) when rs56113850 was added to the model; when an interaction term (rs56113850*rs113288603) was also added to the model the effect size further increased (*beta* = -0.62, p = 1.32E-03). Similarly, the effect size of rs56113850 increased from -0.67 (p<2E-16) to -0.83 (p<2E-16) when rs113288603 was added to the model; addition of an interaction term (rs56113850*rs113288603) to the model did not further affect the results (*beta* = -0.82, p<2E-16).

According to LD estimation in the large FINRISK sample (N = 19857), rs56113850 and rs12461964 share an LD block with the known reduced activity allele CYP2A6*2, and esv2663194 shares a block with the known reduced activity allele CYP2A6*9, while rs113288603 is located outside these LD blocks (S5 Fig). Based on pairwise LD values both CYP2A6*2 and CYP2A6*9 exhibit strong LD with all four independent SNPs (S4 Table), and after conditioning on the top-SNP (rs56113850) neither were genome-wide significant (S5 Table).

Age, sex, and BMI explained altogether 8.9%, 6.1%, and 0.53% of the variance in NMR in FinnTwin, YFS, and FINRISK2007 samples, respectively. The top-SNP rs56113850 alone explains 14–23% of the variance in NMR in the three cohorts (Table 2). Further, the percentage of variance explained by the three independent SNPs (rs56113850, rs113288603, and esv2663194) when jointly included in the model was 20.8% in FinnTwin, 31.4% in YFS, and 26.3% in FINRISK (increasing to 27.7% when rs12461964 was included).

In the wGRS analyses highly similar results were obtained for the 3-SNP and 4-SNP wGRSs; only results for the 4-SNP wGRS are presented. For ease of interpretation, the wGRS was constructed as a weighted sum of major alleles (all of which increase nicotine clearance rate). In the wGRS meta-analysis of 3954 current smokers statistically significant association was detected for quantitative CPD (*beta* = 0.10, 95% CI 0.04–0.16, p = 0.0019), suggesting that individuals with faster metabolism smoke more. The wGRS meta-analysis for current (N = 3954) vs. former (N = 3543) smoking showed association with increased likelihood of being a former smoker (OR = 1.39, 95% CI 1.09–1.76, p = 0.007). Similarly, the major allele of CYP2A6*2 showed a trend of association with increased likelihood of being a former smoker (OR = 1.03, 95% CI 0.72–1.47, p = 0.89), although the results were not statistically significant. To scrutinize potential confounders for cessation, we utilized available questionnaire data and excluded NAG-FIN individuals who either have a DSM-IV major depression disorder diagnosis or report quitting due to adverse health consequences and FINRISK individuals who have a relevant somatic diagnosis, are on disability pension, or rate their health as ‘poor’. Further, we adjusted the analyses for alcohol consumption. After these exclusions, 2946 current and 2591 former smokers remained, and the wGRS result no longer was statistically significant (OR = 1.30, 95% CI 0.95–1.78, p = 0.10). We also tested these models with the rs56113850*rs113288603 interaction term included as a covariate; in all the models the interaction term was non-significant (p>0.05) and so a more complex model was not justified.

In the meQTL analyses of the 719 genome-wide significant SNPs and 158 CpG sites within the target region, methylation values of 16 CpG sites showed statistically significant association with 173 of the SNPs (FDR corrected p<0.05) (S6 Table). Among the highlighted genes were *EGLN2*, *CYP2A7*, *CYP2F1* (*Cytochrome P450*, *Family 2*, *Subfamily F*, *Polypeptide 1*) (ENSG00000197446), and *DLL3* (*Delta-like 3* (*Drosophila*)) (ENSG00000090932) (S7 Table). To distinguish between our hypotheses B (‘SNPs affect NMR via methylation’) and C (‘SNPs affect methylation via NMR’) we performed CIT, and confirmed that methylation at the CpG site tagged by cg08551532 (in *DLL3*) mediates the effect of SNPs on NMR (S8 Table). We detected no evidence supporting hypothesis C.

## Discussion

Nicotine metabolism rate is one of the key factors affecting smoking behavior, and has also been shown to contribute to the efficacy of cessation pharmacotherapy [22]. Many smokers find it exceedingly difficult to succeed in quitting, even when they have a major cardiovascular illness and smoking cessation would greatly improve their prognosis [53]. Current pharmacotherapies and behavioral counselling enhance smoking cessation rate on average by only approximately 2-fold [54], highlighting the need for more effective cessation support. Unraveling the genetic architecture of nicotine metabolism may enhance development of tailored smoking cessation pharmacotherapies.

MZ and DZ twins of FinnTwin12 and FinnTwin16 cohorts yielded a heritability estimate of 0.81 for NMR, confirming that genetic effects are major determinants of inter-individual variance in NMR. This estimate is higher than previous estimates obtained in an experimental setting [16], perhaps due to less heterogeneity in the Finnish sample. Our aim was to identify novel genetic variants affecting nicotine metabolism. We utilized NMR, a biomarker of nicotine metabolism, in a GWAS meta-analysis of three Finnish cohorts, and identified association on 19q13.2. This locus harbours a number of genes, including several members of the cytochrome P450 gene family. Three independent genome-wide significant signals were detected, all located either within or in the immediate vicinity of *CYP2A6*, the gene encoding the main metabolic enzyme for nicotine. A fourth independent signal in *CYP2A6* emerged in one of the samples (FINRISK2007); however, this was not seen in the meta-analysis. The minor alleles of all of the independent variants associated with decreased NMR values, *i*.*e*. decreased nicotine clearance rate. All the independent variants are novel signals and have not been previously been highlighted in any smoking-related GWAS.

Our top-SNP (rs56113850) is located in intron 4 of *CYP2A6*, has a high minor allele frequency (MAF_FINRISK_ = 0.44) and a prominent effect size (*beta* = -0.65), and alone accounts for a substantial percentage of variance (14–23%) in NMR in the three Finnish cohorts. Our second independent SNP (rs113288603) is located 5.9 kb upstream of *CYP2A6*, seems to be enriched in the Finnish population (MAF_FINRISK_ = 0.15 vs. MAF_EUR_ = 0.09), has a small effect size (*beta* = -0.02), and accounts for less than 1% of variance in NMR in the three cohorts. Neither of these variants has predicted functional consequences. Interestingly, our data support a plausible interplay between rs113288603 and rs56113850, as the effect sizes significantly increase when the other SNP is added to the model. These findings are in line with our GWAS data, as rs113288603 shows no association in any of the cohort-specific GWAS or in the meta-analysis, but in an analysis conditioned on rs56113850 it emerges as the second independent genome-wide significant SNP. The plausible mechanism underlying the interplay remains to be determined.

Our third independent variant (esv2663194) tags a 32 kb deletion that affects both *CYP2A6* and *CYP2A7*. Esv2663194 has a low minor allele frequency (MAF_FINRISK_ = 0.03) and a prominent effect size (*beta* = -1.08), and accounts for 3–8% of variance in NMR in the three cohorts. The 32 kb (chr19:41355715–41387669, according to GRCh37/hg19) deletion abolishes exons 1–2 in *CYP2A6* and exons 2–9 in *CYP2A7* when compared to the reference sequence; both genes have multiple isoforms and the consequence of the deletion varies between the isoforms. The 32 kb deletion may produce a similar construct as CYP2A6*12, which is a hybrid allele formed by an unequal crossover between *CYP2A6* and *CYP2A7*. CYP2A6*12 is composed of the 5′-regulatory region and exons 1–2 of *CYP2A7* and exons 3–9 and the 3′-regulatory region of *CYP2A6*, and harbors 10 amino acid differences when compared to the wild-type *CYP2A6* allele, with an allele frequency of 2.2% reported among Spaniards [51]. CYP2A6*12 is shown to have reduced enzyme activity *in vivo* [55, 56]; in line with this, the minor allele of esv2663194 associates with decreased clearance rate. Further studies are needed to confirm whether the 32 kb deletion tagged by esv2663194 indeed creates a construct with properties similar to CYP2A6*12.

Our fourth independent SNP (rs12461964; detected in the FINRISK2007 sample) is located 8.2 kb downstream of *CYP2A6*, has a high minor allele frequency (MAF_FINRISK_ = 0.45) and a prominent effect size (*beta* = -0.61). Rs12461964 is in high LD with the top-SNP rs56113850 (D’ = 0.85); although it was identified as an independent SNP in conditional analyses in the FINRISK2007 sample, the detected association likely reflects LD with rs56113850.

To date, the most widely studied *CYP2A6* variants include CYP2A6*2 (rs1801272, L160H) which encodes a catalytically inactive enzyme, a whole gene deletion allele CYP2A6*4, CYP2A6*9 (rs28399433, -48T>G), which has an alteration in the TATA box resulting in lower expression of a structurally normal protein, and CYP2A6*12 discussed above [13]. Of these characterized reduced-activity variants, our data included CYP2A6*2 and CYP2A6*9, both of which showed genome-wide significant association but were not identified as independent signals in conditional analyses. A previous very large (N = 85997) meta-analysis of self-reported CPD showed genome-wide significant association on 19q13.2, with strongest evidence obtained for rs4105144, which is in LD with CYP2A6*2 (D′ = 1.0 in CEU) [14]. Similarly, LD between our novel independent signals and both CYP2A6*2 and CYP2A6*9 was high (D’ = 0.89–1.00). The percentage of variance in NMR explained by CYP2A6*2 and CYP2A6*9 in our study cohorts was up to 6% and 12%, respectively. A previous study using an ethnically diverse sample suggested that 15–20% of variance in NMR is accounted for by the four characterized reduced-activity variants (CYP2A6*2, CYP2A6*4, CYP2A6*9, and CYP2A6*12) [16]; much of the genetic variation would not have been tested for in this study (i.e. CYP2A6*10 –*35). Interestingly, in our study CYP2A6*9 alone explained a large fraction of variance in NMR, possibly due to the higher minor allele frequency in the Finnish cohorts (MAF 0.12–0.14) compared to that reported in the Caucasian population (MAF_EUR_ = 0.07). In addition to CYP2A6*2 and CYP2A6*9, our data set included three additional known alleles, CYP2A6*14 (rs28399435; S29N), CYP2A6*18 (rs1809810; Y392F), and CYP2A6*21 (rs6413474; K476R). Although they are non-synonymous variants, their effect on nicotine clearance likely is minimal. CYP2A6*14 does not appear to affect enzyme activity [57, 58], and although CYP2A6*18 has a decreased activity towards another substrate, coumarin, activity towards nicotine is unaffected [59]. Based on *in vivo* studies, CYP2A6*21 showed normal activity in a Caucasian population [60]. After conditioning on our top-SNP (rs56113850), none of the five known alleles were genome-wide significant, suggesting that rs56113850 captures information on all of these alleles.

The percentage of variance explained by our novel independent SNPs was very high (up to 31%), exceeding the estimates for the four functional variants frequently genotyped in Caucasians [16]. Our novel variants likely tag multiple functional variants, both known and unidentified ones, and thus capture information on relevant haplotypes. The population-attributable effect of the novel independent variants thus is far greater than that of the known functional variants, and for the purpose of estimating the rate of nicotine metabolism our novel SNPs are more informative than the known previously characterized reduced-activity variants.

Based on our twin modelling, the heritability of NMR is 0.81. Although our novel independent SNPs capture a strikingly large fraction of this, major fraction still remains unaccounted for. This may be due to limitations of GWAS with lack of coverage of *CYP2A6* duplications, translocations, and rare variants. Further, the high sequence homology between *CYP2A6*, *CYP2A7*, and *CYP2A13* may prohibit detection of variants within homologous regions. SNP genotyping technologies using short probes will not be able to detect these variants with high specificity; most likely such variants will fail the HWE threshold, and thus will be excluded from analyses. In addition, limitations of statistical power result in inability to detect all relevant signals. In our GWAS meta-analysis we had high power to detect signals with common SNPs (MAF >5%) that have medium to high effect sizes. This is reflected in our top-SNPs, three of which are not only common but also have large effect sizes, and one that is rare (MAF = 0.03) but has a large effect size (beta = -1.08). We had very low power to detect signals with rare SNPs that have low effect sizes, and it is likely that we missed those signals; larger studies are needed to capture these signals.

Ethnicity has been reported as a prominent factor affecting NMR; however, all the subjects in the current study were Caucasian (Finns). Age, sex, and BMI accounted for 8.9% and 6.1% of variance in NMR in the FinnTwin and YFS samples, respectively. This is in line with a previous study showing that various non-genetic factors account for altogether 8% of inter-individual variance in NMR [17]. However, in the FINRISK2007 sample age, sex, and BMI only accounted for 0.5% of variance in NMR, which may be due to older age and longer overall duration of smoking with more cessation than among the young adult FinnTwin and YFS samples.

Although only variants in *CYP2A6* were highlighted as independent signals, other interesting genes, such as *CYP2B6*, *CYP2A7*, *EGLN2*, and *NUMBL*, reside within the 19q13 locus, and showed genome-wide significant association in our GWAS meta-analysis. Our top-SNP in *CYP2B6* (rs7260329) has been highlighted in the large GWAS meta-analysis of CPD [14]. CYP2B6 can also catalyze nicotine metabolism to cotinine and to nornicotine [9]; the *CYP2B6* gene sits adjacent to *CYP2A6* and they share some common regulation. CYP2B6 also metabolizes several other drugs of abuse, as well as bupropion, an atypical antidepressant also used as a smoking cessation aid [5]. Several functional *CYP2B6* variants have been identified. The most prevalent and clinically important variant is CYP2B6*6, characterized as a haplotype consisting of two linked non-synonymous variants CYP2B6*4 (rs2279343 (K262R)) and CYP2B6*9 (rs3745274 (Q172H)), resulting in a splice site variant with reduced function [61]. In the current study the non-synonymous SNPs defining CYP2B6*4, CYP2B6*9, as well as CYP2B6*5 (rs3211371 (R487C)) did not show genome-wide significant results, but were in high LD with the top-SNP in *CYP2B6* (rs7260329) (S9 Fig). Further, rs7260329 shows modest LD with our independent SNPs rs56113850 (D’ = 0.40), esv2663194 (D’ = 0.65), and rs12461964 (D’ = 0.43), but not with rs113288603 (D’ = 0.04); thus, it is unclear whether the detected association simply reflects LD with the independent variants residing in *CYP2A6*.

The interpretation of the detected *CYP2A7* association is challenging, as the possible role of CYP2A7 in nicotine metabolism is unknown. Our top SNP in *CYP2A7* (rs28602288) is located within a predicted promoter region. It is plausible that the association between rs28602288 and NMR reflects the regulation of CYP2A6*12 (a hybrid allele of *CYP2A6* and *CYP2A7*) or similar constructs, such as the one generated by the 32 kb deletion tagged by esv2663194, rather than implies a role for *CYP2A7* in nicotine clearance. Rs28602288 is in LD with all of the independent variants (D’ = 0.67–1.00).

Two additional interesting genes were highlighted. *Egl-9 Family Hypoxia-Inducible Factor 2 (EGLN2)* is a key component of the oxygen-sensing pathway that regulates the expression of various downstream genes, and responses e.g. to carbon monoxide (CO) and cigarette smoke exposure. In a recent study, *EGLN2* was associated with CPD and breath CO independent of *CYP2A6*, although it was not associated with nicotine metabolism [62]. The top-SNP in *EGLN2* (rs76443752) is in LD with all of the independent *CYP2A6* variants (D’ = 0.67–1.00). Another potentially interesting candidate is *Numb Homolog (Drosophila)-Like (NUMBL)* which encodes a protein that maintains progenitor cells during cortical neurogenesis [63] and has previously been implicated in lung cancer [64, 65]. The top-SNP in *NUMBL* (rs4802082) is in LD with all of the independent variants (D’ = 0.48–0.96).

We constructed wGRS using the independent 19q13.2 SNPs and tested whether the wGRS predicts smoking behavior in two independent Finnish samples. The wGRS constructed of major alleles (increasing the metabolism rate) was associated with increased smoking quantity. This is in line with previous evidence of faster metabolizers smoking more [66]. Adding the rs56113850*rs113288603 interaction term to the wGRS analyses did not improve model fit, suggesting that the simpler model with main effects was sufficient. Our unadjusted wGRS results for current vs. former smoking suggested that major alleles that associate with faster metabolism associate with increased odds of being a former smoker; after adjustment and exclusion for potential confounders the wGRS result no longer was significant. Although the sample size decreased, the confidence intervals did not significantly increase, suggesting that the change in results is due to reduced confounding rather than reduced power. Many clinical trials and epidemiological studies indicate that slow metabolizers quit more often than normal metabolizers [20, 67, 68]. Possibly our wGRS does not capture all the relevant aspects of measured NMR or the cross-sectional nature of the FINRISK smoking status data did not permit us to fully replicate earlier findings.

We followed up the 719 genome-wide significant SNPs by meQTL analyses in order to annotate their potential functional consequence. As smoking is known to induce significant changes in methylation patterns [69], only cotinine-verified current smokers (cotinine≥10ng/ml) were included in the analyses. Several SNPs showed significant association with methylation values of 16 CpG sites located within the target region. The 16 CpG sites overlap with relevant genes, such as members of the cytochrome P450 gene family (*CYP2F1* and *CYP2A7*) and *EGLN2*. According to CIT, methylation in one CpG site mediate the effect of SNPs on the variance observed in NMR. This CpG site (tagged by cg08551532) is located in *DLL3*, which is involved in neurogenesis via its role in the Notch signaling pathway [70]. Further, *DLL3* has been shown to be silenced by methylation in human hepatocellular carcinoma (HCC), leading to restricted growth of cancer cells [71]. Expression of *NOTCH3*, encoding for a receptor for DLL3, has been shown to be influenced by cigarette smoke [72]. The potential mechanism how *DLL3* may affect nicotine metabolism remains to be determined.

To our knowledge this is the first GWAS of NMR reported to date. Despite a relatively small sample size (N = 1518), we detected a multitude of genome-wide significant signals on 19q13.2, highlighting the power of informative biomarkers in GWAS and demonstrating the power of Finnish population samples to identify novel genes even with a modest sample size. Our genetic and epigenetic analyses enclosed several genes as potential players in regulating NMR. Four members of the cytochrome P450 gene family (*CYP2A6*, *CYP2B6*, *CYP2A7*, and *CYP2F1*) were highlighted, although only *CYP2A6* encompassed independent signals. Three additional highlighted genes (*DLL3*, *NUMBL*, and *EGLN2*) are functionally linked according to the GeneMania database (http://www.genemania.org/), suggesting that an interplay between these genes may influence NMR. Future studies are needed to elucidate the potential role of these genes in NMR. The detected novel *CYP2A6* variants explain a strikingly large fraction of variance (up to 31%) in NMR in the current sample, suggesting that they tag both known and unidentified functional variants. The population-attributable effect of the detected independent variants is thus far greater than that of the four functional variants frequently genotyped in Caucasians (CYP2A6*2, *4, *9, and *12). Further, we enclose evidence for plausible epigenetic mechanisms on 19q13.2 influencing NMR.

## Supporting Information

S1 Fig Distribution of nicotine metabolite ratio (NMR) before and after transformation. (A) Distribution of NMR in the FinnTwin sample, (B) distribution of rank transformed NMR in the FinnTwin sample, (C) distribution of NMR in the YFS sample, (D) distribution of rank transformed NMR in the YFS sample, (E) distribution of NMR in the FINRISK2007 sample, (F) distribution of rank transformed NMR in the FINRISK2007 sample.
(TIF)

S2 Fig Interquartile range (IQR) of normalized methylation beta values among 1424 CpG sites within the 19q13.2 target region (chr19:39046965–44210562, according to GRCh37/hg19). A total of 158 probes exceeded the threshold of interquartile range ≥0.05 and were selected for mQTL analyses.
(TIF)

S3 Fig LD structure of *CYP2A6* with ±10 kb flanking regions. (A) Pairwise D’, (B) Pairwise R^2^. Block boundaries were defined by the ‘solid spine of LD’ option of Haploview [45].
(TIF)

S4 Fig LD structure of *CYP2A6* (genome-wide significant SNPs only). (A) Pairwise D’, (B) Pairwise R^2^. Block boundaries were defined by the ‘solid spine of LD’ option of Haploview [45].
(TIF)

S5 Fig LD structure of *CYP2A6* (independent genome-wide significant SNPs and potential functional SNPs regardless of their p-value). (A) Pairwise D’, (B) Pairwise R^2^. The second independent SNP (rs113288603) is also included, although it was not genome-wide significant in the GWAS meta-analysis but only in analyses conditioned on the top-SNP (rs56113850). Block boundaries were defined by the ‘solid spine of LD’ option of Haploview [45].
(TIF)

S6 Fig Distribution of NMR and rank transformed NMR values among different genotype groups of the four independent SNPs and the five known *CYP2A6* alleles in the Young Finns Study (YFS) sample (N = 714). The exact number of subjects in each comparison varies depending on the number of subjects with non-missing data for that SNP. Number of subjects is indicated for each genotype group.
(TIF)

S7 Fig LD structure of *CYP2B6*. (A) Pairwise D’, (B) Pairwise R^2^. Block boundaries were defined by the ‘solid spine of LD’ option of Haploview [45].
(TIF)

S8 Fig LD structure of *CYP2B6* (genome-wide significant SNPs only). (A) Pairwise D’, (B) Pairwise R^2^. Block boundaries were defined by the ‘solid spine of LD’ option of Haploview [45].
(TIF)

S9 Fig LD structure of *CYP2B6* (the top-SNP and potential functional SNPs regardless of their p-value). (A) Pairwise D’, (B) Pairwise R^2^. Rs2279343 (CYP2B6*4) was not available in the FINRISK data used for LD calculations. Block boundaries were defined by the ‘solid spine of LD’ option of Haploview [45].
(TIF)

S1 Table Estimated power of the meta-analysis sample (N = 1518) to detect signals at the p<5E-08 significance threshold and LD(r^2^) = 0.8 between the causal allele and the marker allele, with a range of MAFs and effect sizes. Effective sample size was N = 1486 after accounting for interclass correlation within the 78 DZ pairs included in the FinnTwin sample.
(XLSX)

S2 Table Cohort-specific GWAS and meta-analysis results for the 719 genome-wide significant SNPs on 19q13.2. (XLSX)

S3 Table Cohort-specific GWAS and meta-analysis results for all *CYP2A6* SNPs included in our data set. (XLSX)

S4 Table Linkage disequilibrium (D’ and R^2^) between the four independent SNPs and the five characterized *CYP2A6* alleles included in the data set. (XLSX)

S5 Table Comparison of results obtained from GWAS meta-analysis and analysis conditioned on the top-SNP (rs56113850) for the independent SNPs and for the five characterized *CYP2A6* alleles included in the data. (XLSX)

S6 Table Methylation quantitative trait loci (meQTL) results. Analyses were done for 158 CpG sites and 719 SNPs on 19q13.2. Results are shown only for the 16 CpG sites for which an FDR corrected p-value below 0.05 (highlighted) was obtained. The 16 CpG sites have 173 SNPs significantly associating with them.
(XLSX)

S7 Table Annotation of the 16 CpG sites highlighted in meQTL. (XLSX)

S8 Table Causal inference test results shown for one CpG site (cg08551532 in *DLL3*) in which methylation mediates the effect of SNPs on NMR. The other 15 CpG sites highlighted in meQTL analyses did not show evidence of mediation.
(XLSX)
