# Supplementary figures and images for: A Genome-Wide Association Study of a Biomarker of Nicotine Metabolism
Source: PLoS Genet. 2015 Sep 25;11(9):e1005498. doi: 10.1371/journal.pgen.1005498 (PMC4583245; doi:10.1371/journal.pgen.1005498)

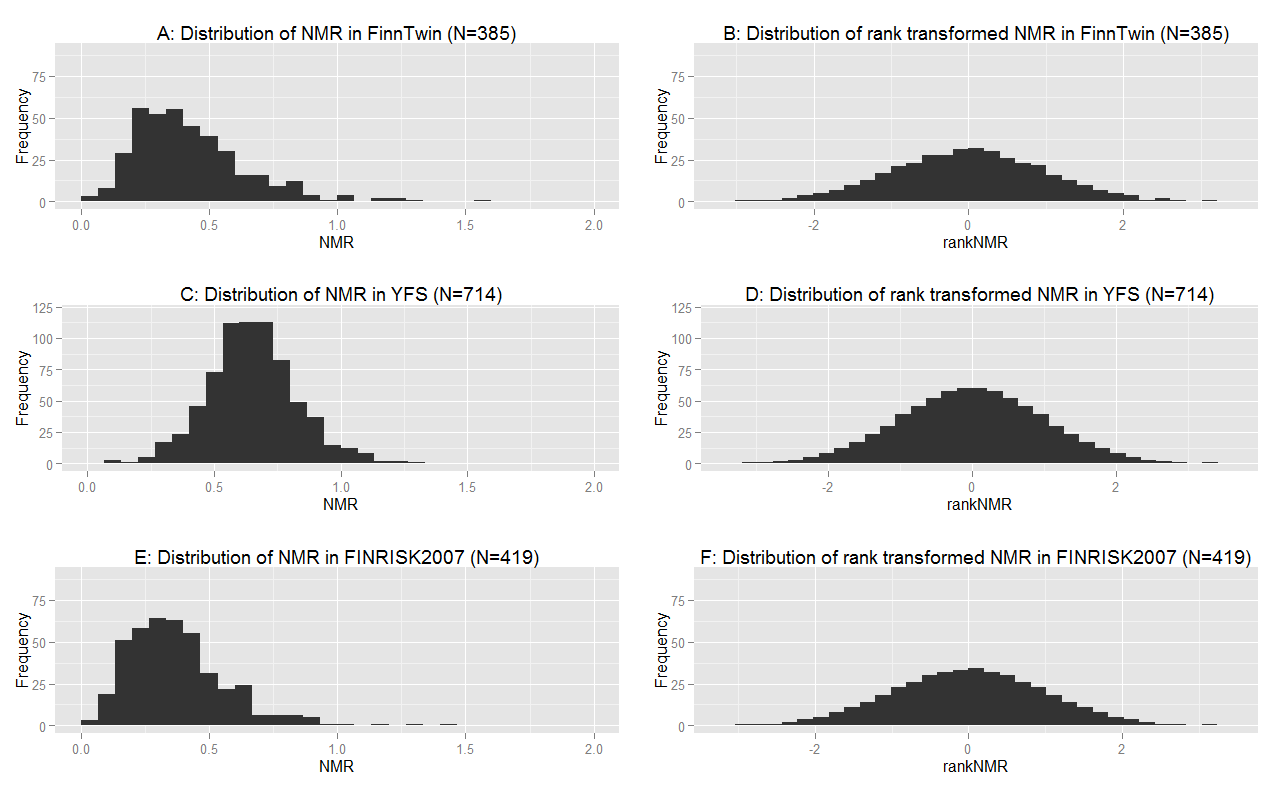

Supplement: S1 Fig — (A) Distribution of NMR in the FinnTwin sample, (B) distribution of rank transformed NMR in the FinnTwin sample, (C) distribution of NMR in the YFS sample, (D) distribution of rank transformed NMR in the YFS sample, (E) distribution of NMR in the FINRISK2007 sample, (F) distribution of rank transformed NMR in the FINRISK2007 sample. (TIF) [file pgen.1005498.s001.tif]

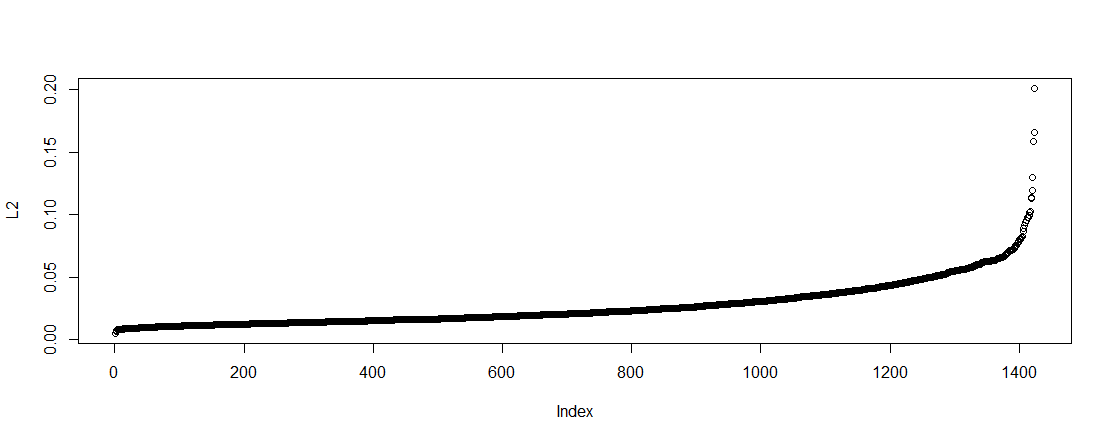

Supplement: S2 Fig — A total of 158 probes exceeded the threshold of interquartile range ≥0.05 and were selected for mQTL analyses. (TIF) [file pgen.1005498.s002.tif]

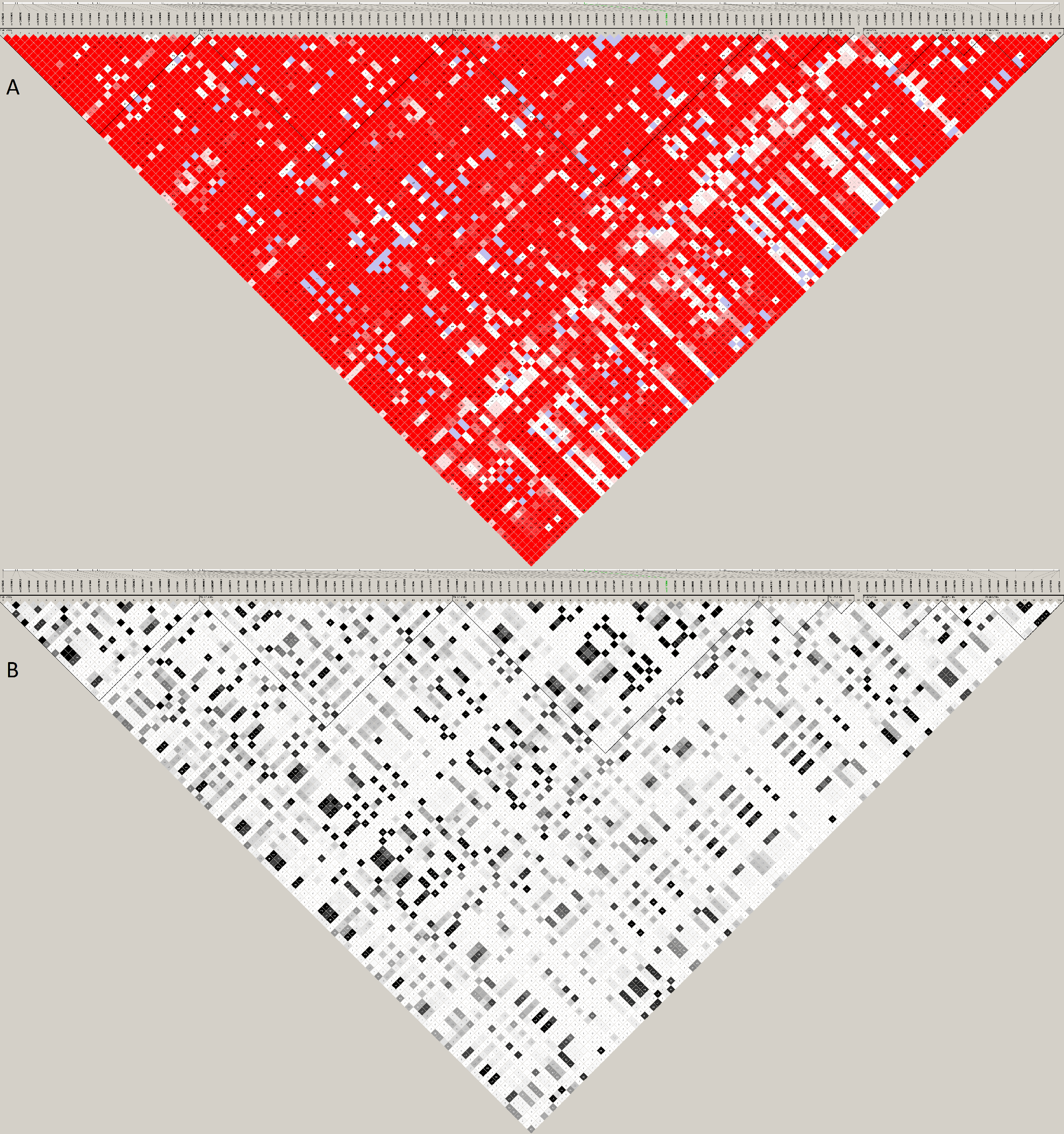

Supplement: S3 Fig — (A) Pairwise D’, (B) Pairwise R2. Block boundaries were defined by the ‘solid spine of LD’ option of Haploview [45]. (TIF) [file pgen.1005498.s003.tif]

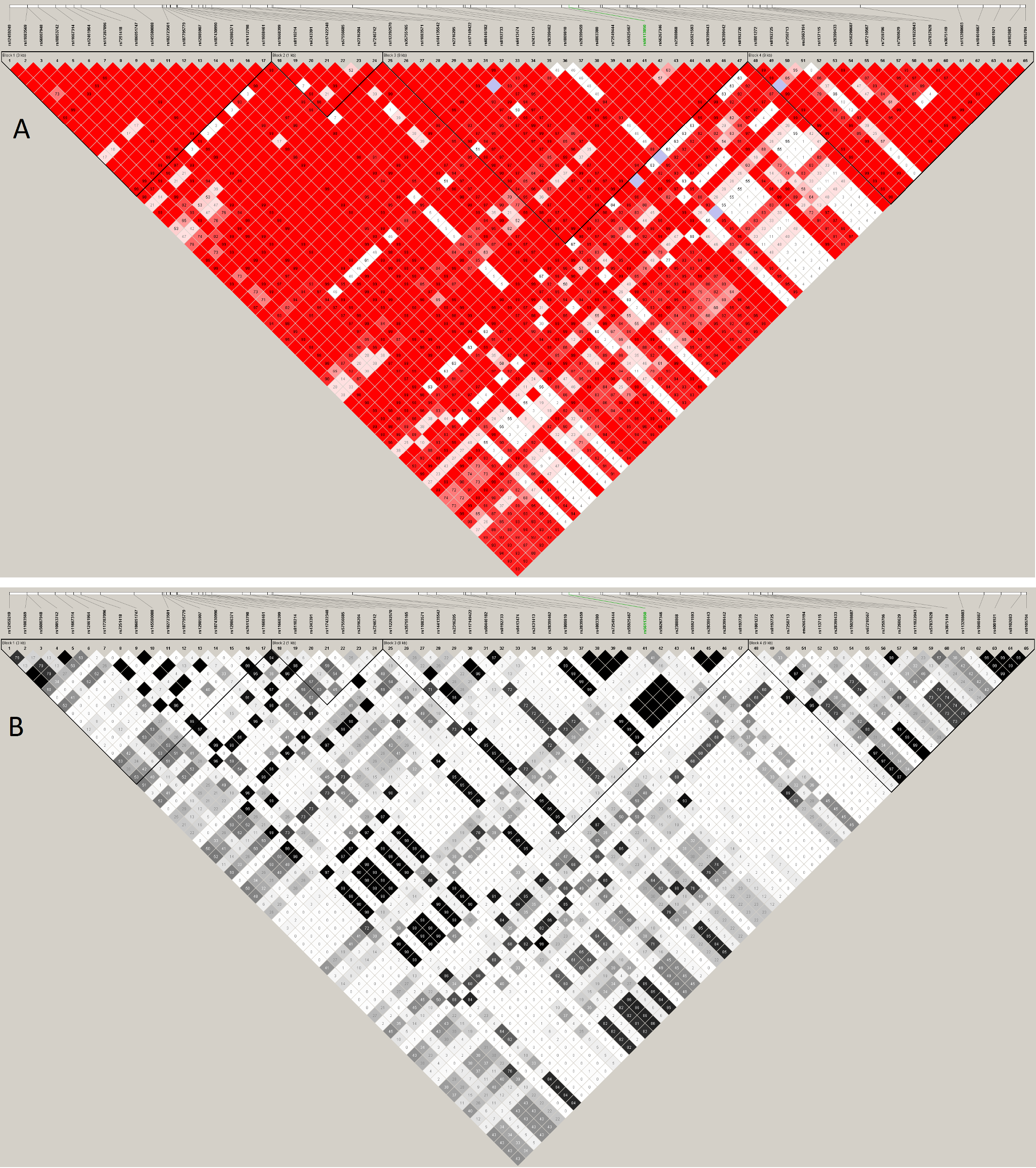

Supplement: S4 Fig — (A) Pairwise D’, (B) Pairwise R2. Block boundaries were defined by the ‘solid spine of LD’ option of Haploview [45]. (TIF) [file pgen.1005498.s004.tif]

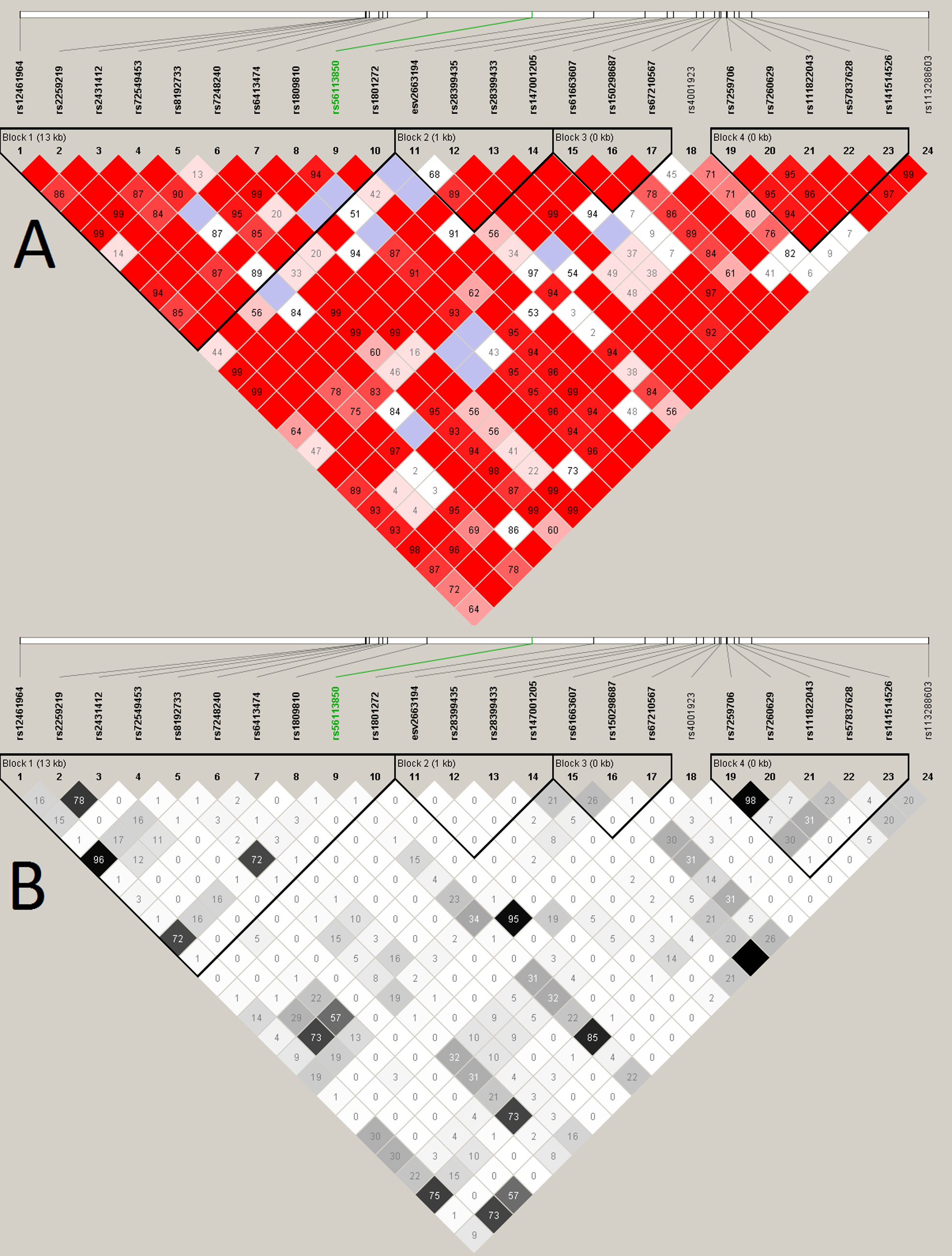

Supplement: S5 Fig — (A) Pairwise D’, (B) Pairwise R2. The second independent SNP (rs113288603) is also included, although it was not genome-wide significant in the GWAS meta-analysis but only in analyses conditioned on the top-SNP (rs56113850). Block boundaries were defined by the ‘solid spine of LD’ option of Haploview [45]. (TIF) [file pgen.1005498.s005.tif]

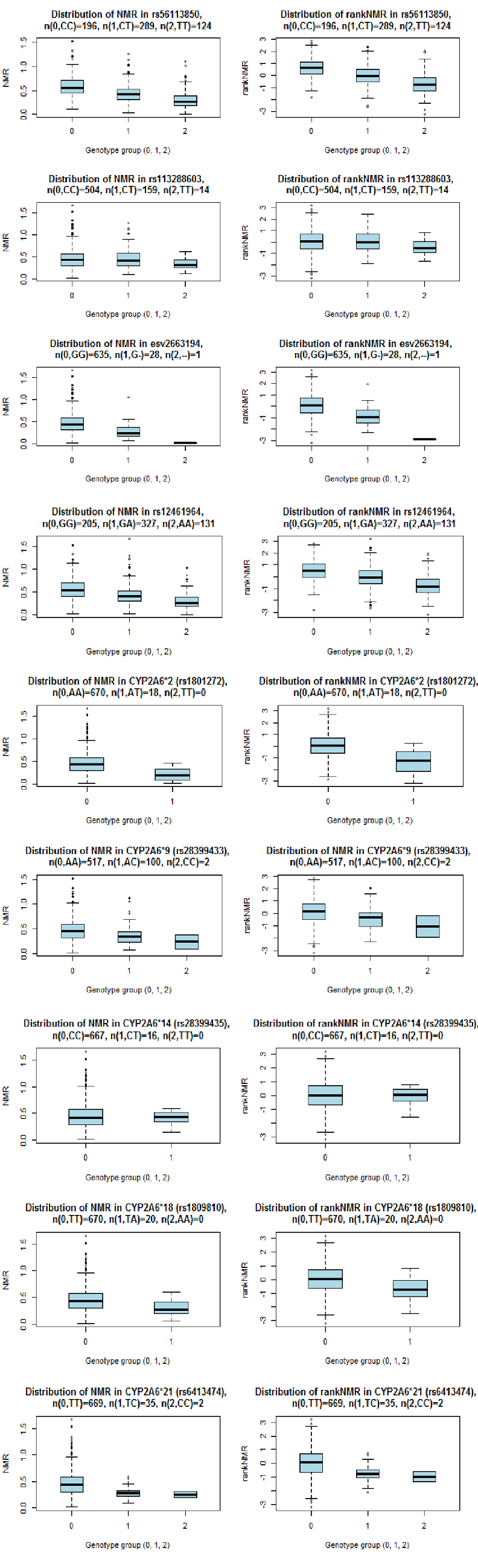

Supplement: S6 Fig — The exact number of subjects in each comparison varies depending on the number of subjects with non-missing data for that SNP. Number of subjects is indicated for each genotype group. (TIF) [file pgen.1005498.s006.tif]

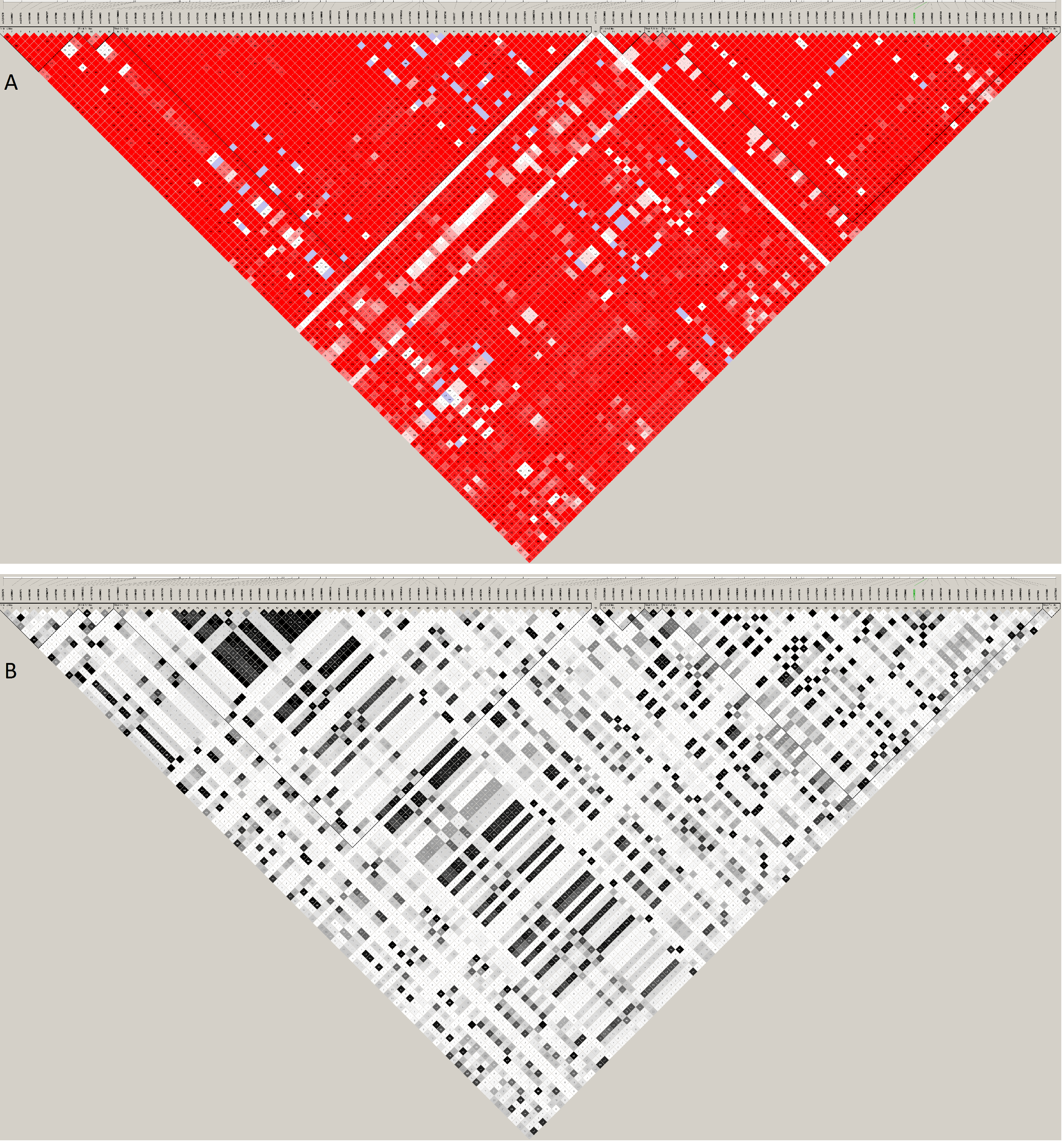

Supplement: S7 Fig — (A) Pairwise D’, (B) Pairwise R2. Block boundaries were defined by the ‘solid spine of LD’ option of Haploview [45]. (TIF) [file pgen.1005498.s007.tif]

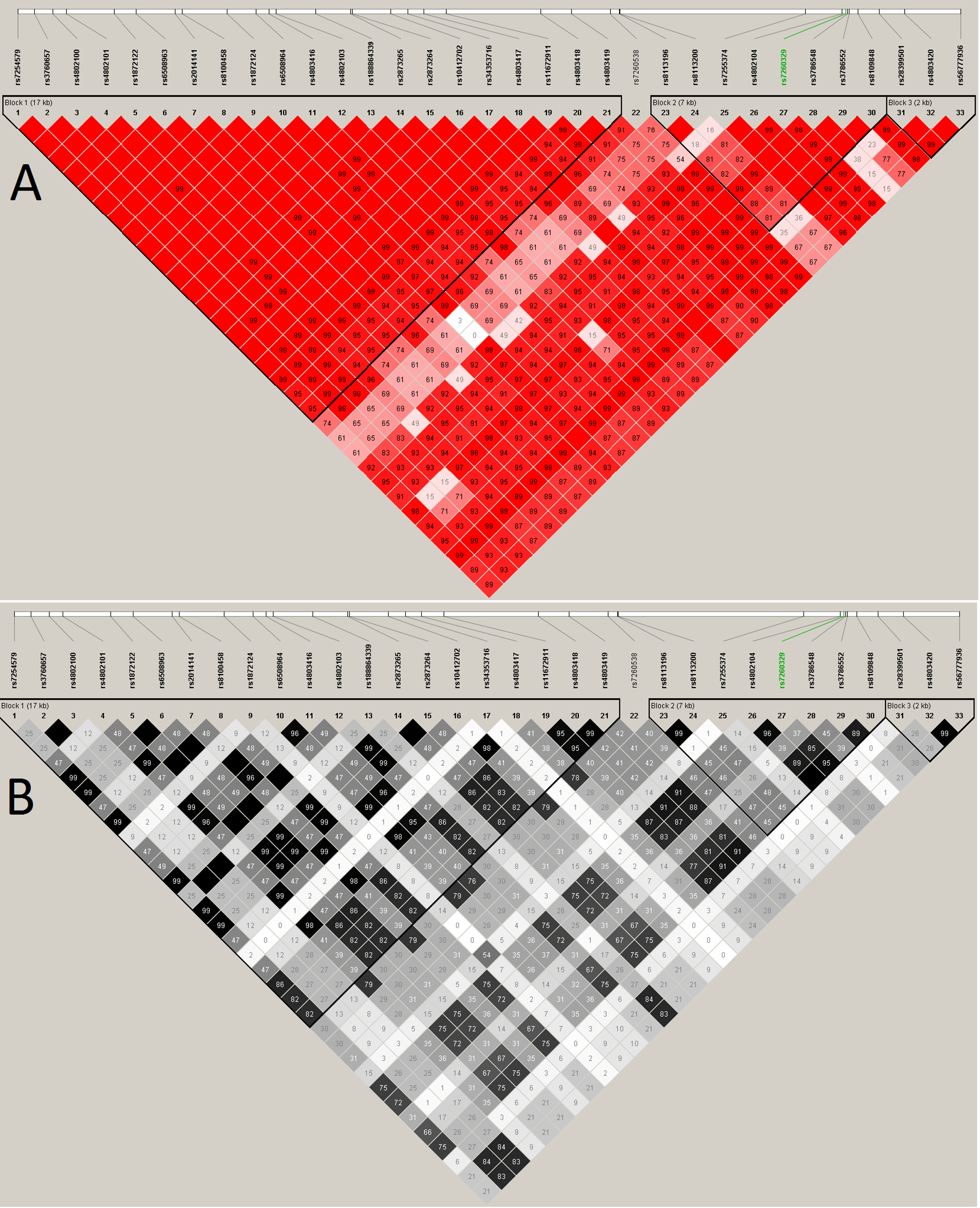

Supplement: S8 Fig — (A) Pairwise D’, (B) Pairwise R2. Block boundaries were defined by the ‘solid spine of LD’ option of Haploview [45]. (TIF) [file pgen.1005498.s008.tif]

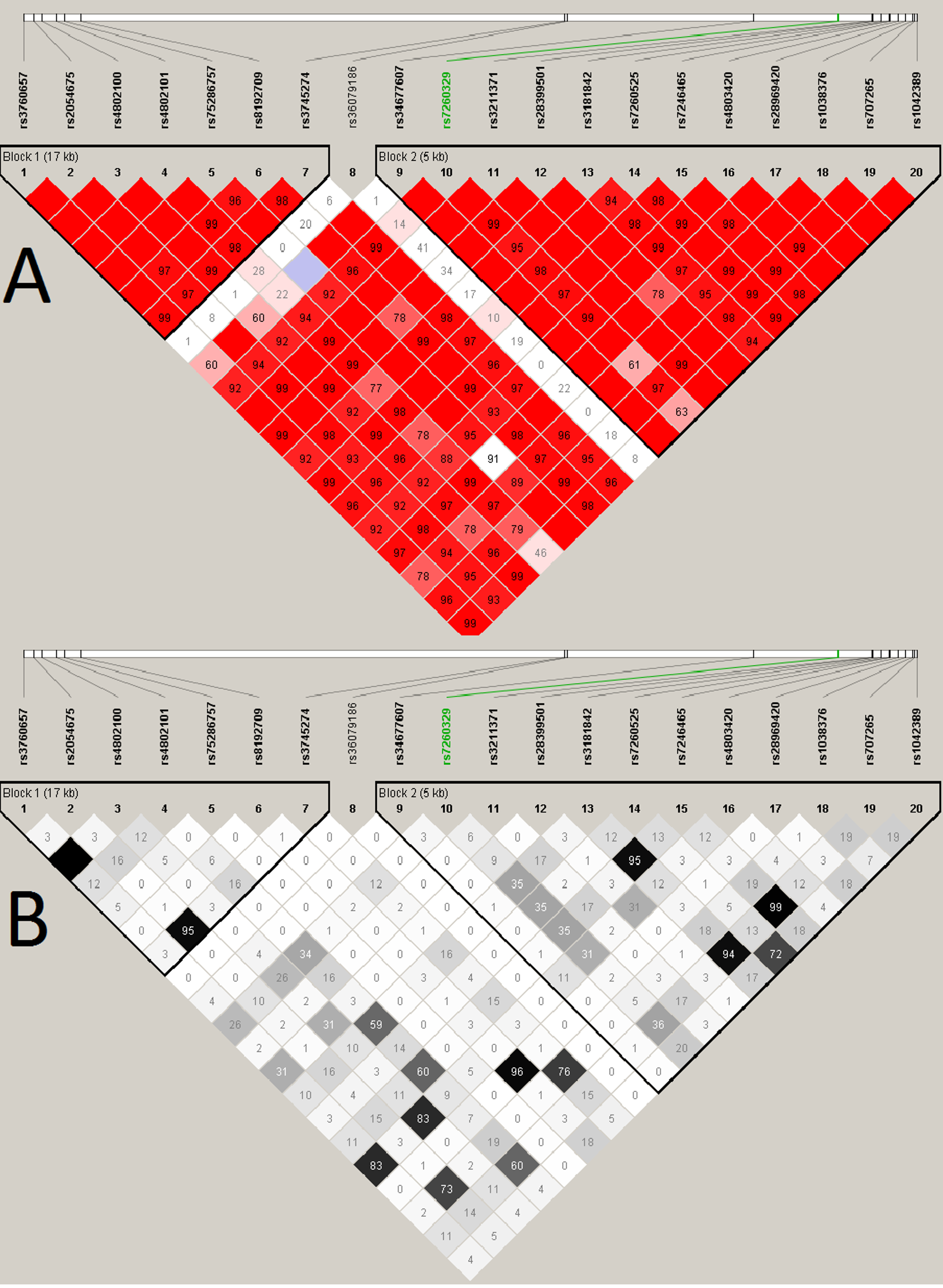

Supplement: S9 Fig — (A) Pairwise D’, (B) Pairwise R2. Rs2279343 (CYP2B6*4) was not available in the FINRISK data used for LD calculations. Block boundaries were defined by the ‘solid spine of LD’ option of Haploview [45]. (TIF) [file pgen.1005498.s009.tif]
